# Inverse expression of survivin and reprimo correlates with poor patient prognosis in gastric cancer

**DOI:** 10.18632/oncotarget.24402

**Published:** 2018-02-05

**Authors:** Paulina Cerda-Opazo, Manuel Valenzuela-Valderrama, Ignacio Wichmann, Andrés Rodríguez, Daniel Contreras-Reyes, Elmer A. Fernández, Gonzalo Carrasco-Aviño, Alejandro H. Corvalán, Andrew F.G. Quest

**Affiliations:** ^1^ Laboratorio de Comunicaciones Celulares, Centro de Estudios en Ejercicio, Metabolismo y Cáncer (CEMC), Programa de Biología Celular y Molecular, Instituto de Ciencias Biomédicas (ICBM), Facultad De Medicina, Universidad de Chile, Santiago, Chile; ^2^ Gastric Cancer Research Group - Laboratory of Oncology, UC Center for Investigational Oncology (CITO), Pontificia Universidad Católica de Chile, Santiago, Chile; ^3^ Advanced Center for Chronic Diseases (ACCDiS), Santiago, Chile; ^4^ Facultad de Ciencias de la Salud, Universidad Central de Chile, Santiago, Chile; ^5^ CIDIE – CONICET - Facultad de Ingeniería, Campus Universitario, Universidad Católica de Córdoba, Córdoba, Argentina; ^6^ Facultad de Ciencias Exactas, Físicas y Naturales, Universidad Nacional de Córdoba, Córdoba, Argentina; ^7^ National Bioinformatics Consortia (BIA) of Argentina, Buenos Aires, Argentina; ^8^ Departamento de Anatomía Patológica, Hospital Clínico José Joaquín Aguirre, Universidad de Chile, Santiago, Chile; ^9^ Core Biodata, Advanced Center for Chronic Diseases (ACCDiS), Santiago, Chile

**Keywords:** gastric cancer, survivin, reprimo, TCGA

## Abstract

**BACKGROUND:**

The objective of the study was to determine the relationship between Survivin and Reprimo transcript/protein expression levels, and gastric cancer outcome.

**METHODS:**

*In silico* correlations between an agnostic set of twelve p53-dependent apoptosis and cell-cycle genes were explored in the gastric adenocarcinoma TCGA database, using cBioPortal. Findings were validated by regression analysis of RNAseq data. Separate regression analyses were performed to assess the impact of p53 status on Survivin and Reprimo. Quantitative reverse-transcription PCR (RT-qPCR) and immunohistochemistry confirmed *in silico* findings on fresh-frozen and paraffin-embedded gastric cancer tissues, respectively. Wild-type (AGS, SNU-1) and mutated p53 (NCI-N87) cell lines transfected with pEGFP-Survivin or pCMV6-Reprimo were evaluated by RT-qPCR and Western blotting. Kaplan-Meier method and Long-Rank test were used to assess differences in patient outcome.

**RESULTS:**

cBioPortal analysis revealed an inverse correlation between Survivin and Reprimo expression (Pearson’s r= −0.3, Spearman’s ρ= −0.55). RNAseq analyses confirmed these findings (Spearman’s ρ= −0.37, p<4.2e-09) and revealed p53 dependence in linear regression models (p<0.05). mRNA and protein levels validated these observations in clinical samples (p<0.001). *In vitro* analysis in cell lines demonstrated that increasing Survivin reduced Reprimo, while increasing Reprimo reduced Survivin expression, but only did so in p53 wild-type gastric cells (p<0.05). Survivin-positive but Reprimo-negative patients displayed shorter overall survival rates (p=0.047, Long Rank Test) (HR=0.32; 95%IC: 0.11-0.97; p=0.044).

**CONCLUSIONS:**

TCGA RNAseq data analysis, evaluation of clinical samples and studies in cell lines identified an inverse relationship between Survivin and Reprimo. Elevated Survivin and reduced Reprimo protein expression correlated with poor patient prognosis in gastric cancer.

## INTRODUCTION

Gastric cancer is the third leading cause of cancer-related deaths worldwide, with 951,000 new cases and 723,000 deaths in 2012 [1]. In molecular terms, many different types of gastric cancer have been described and only more recently has a comprehensive molecular characterization of primary tumors by The Cancer Genome Atlas (TCGA) Research Network [2] begun to shed light on this heterogeneity by segregating cases into four molecular subtypes: (i) tumors positive for Epstein-Barr virus (EBV)-associated gastric carcinoma, (ii) microsatellite-unstable tumors, (iii) genomically stable tumors and (iv) tumors with chromosomal instability [2]. Interestingly, genomically stable tumors are enriched for Lauren’s class diffuse-type gastric cancer and recurrent CDH1, as well as RHOA mutations. On the other hand, tumors with chromosomal instability are enriched for Lauren’s class intestinal-type gastric cancer, with frequent mutations in the tumor protein p53 (TP53), and located at the gastroesophageal junction [2]. In gastric cancer, as in many cancers, deregulation in the expression of cell cycle and apoptosis-related genes, as well as loss of functional p53 play an important role in disease development and progression. Here, we specifically focused our analysis on two cell cycle/apoptosis proteins Survivin (BIRC5) and Reprimo (RPRM).

Survivin is a member of the inhibitor-of-apoptosis protein (IAP) family (reviewed in Garg et al. [3], with multiple physiological and pathological roles that are important in development, metabolism, cell communication, angiogenesis and motility [4].

The Survivin protein is readily detectable in normal gastric mucosa and is proposed there to have a protective function, given that infection with *H. pylori* leads to loss of Survivin in the gastric epithelium and increased apoptosis in gastric cancer cell lines. Thus, at early stages of infection, Survivin aids in the maintenance of gastric epithelial integrity. Loss of Survivin due to *H. pylori* is predicted to disrupt gastric mucosa homeostasis and contribute significantly to chronic inflammation, which then exacerbates signaling pathways that favor disease onset and progression [5, 6]. Importantly, however, despite early loss of Survivin due to infection, the protein is re- or over-expressed in many human cancers and other inflammatory diseases [7]. Also, in gastric cancer cells, Survivin expression is elevated in tumor samples when compared to surrounding normal tissues [8].

Several transcriptional factors have been described that recognize the Survivin promoter region and control protein expression [9, 10]. Among such transcriptional regulators, the tumor suppressor protein p53 was of interest because it represses Survivin expression [11], and is frequently down-regulated or mutated in cancer [12, 13]. Thus, deregulation of p53 may serve to explain, at least in part, the observed upregulation of Survivin associated with cancer development and progression [14, 15].

Alternatively, RPRM expression promotes cell cycle arrest in the G2/M phase and is transcriptionally up-regulated by p53 [16], in addition to being subject to post-translational modifications [16–18]. Interestingly, RPRM protein expression is reduced in gastric tumor samples when compared with non-tumor adjacent mucosa (NTAM) [19, 20] and overexpression of RPRM induces apoptosis in gastric cancer cells [21]. Indeed, RPRM is now considered a novel class II tumor suppressor gene in gastric cancer because its expression is silenced by promoter region hypermethylation [20], possibly attributable to activity of the virulence factor CagA following *H. pylori* infection [22].

Thus, from the available information, both Survivin and RPRM emerged as being potentially important in gastric cancer, but likely playing essentially opposing roles, particularly in controlling G2/M, suggesting also that their expression should be mutually exclusive. In support of this notion, Survivin and RPRM are suppressed or enhanced, respectively, by p53 [11, 16]. Moreover, Survivin reportedly also reduces p53 expression [23], which, in turn, controls RPRM levels. All together, these observations pointed towards the possibility that Survivin upregulation, as frequently observed in cancer, may contribute to the loss of RPRM.

Since its publication, the TCGA has become a powerful tool to interrogate *in silico* connections between genes and pathways altered in cancer and particularly to identify patterns of mutual exclusion and/or coexistence between genes that are linked to the pathology [24, 25]. Thus, we initiated this study in an unbiased manner by first interrogating the existence of connections between cell cycle and apoptosis in general, and then focusing on Survivin and RPRM. Specifically, we sought to determine whether Survivin and RPRM expression might be mutually exclusive. Initial analysis of RNAseq data from the gastric adenocarcinoma TCGA project [2] revealed that while for many pairs of cell cycle/apoptosis genes co-expression was common, Survivin and RPRM were unique because expression was mutually exclusive and the connection appeared to be p53 dependent. This observation was then validated by PCR analysis in clinical samples. Subsequently, we confirmed, with the help of gastric cancer cell lines, functionality of the connection between Survivin and RPRM. Finally, the clinical significance of the mutual exclusion was evaluated in a large data set of gastric cancer cases, where survival rates for patients with Survivin positive tumors was found to be significantly reduced in those cases where RPRM was absent.

## RESULTS

### Survivin and RPRM expression are inversely correlated in a p53-dependent manner as determined by analysis of *in silico* RNAseq data from TCGA

**Figure 1:**
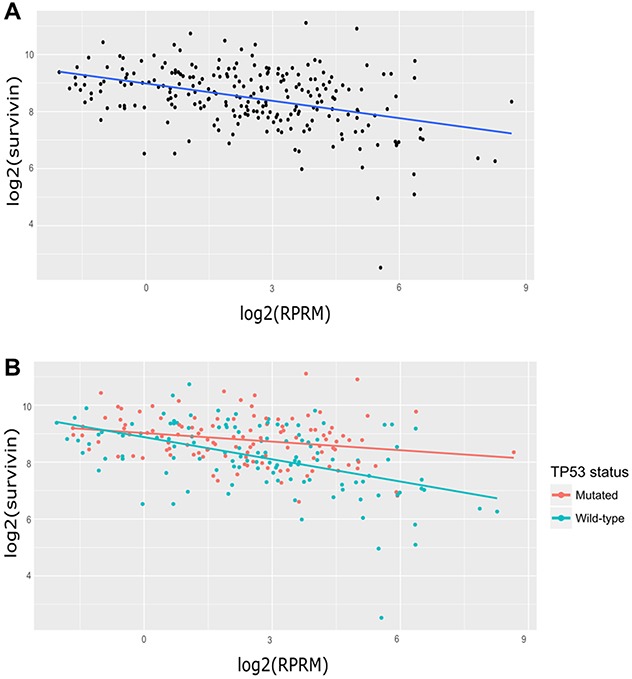
Analysis of Survivin and RPRM transcript expression in gastric cancer samples from the TCGA database **(A)** Linear regression model for Survivin and RPRM in 237 cases for which Survivin and RPRM counts were greater than zero and p53 status was available. *log*2(*Survivin*) = 8.98 − 0.20 ^*^ *log*2(*RPRM*) **(B)** Linear regression models for Survivin and RPRM according to p53 status. For p53-mutated samples (n=116, red regression line): *log*2(*Survivin*) = 9.02106 − 0.10055 ^*^ *log*2(*RPRM*) For wild-type p53 samples (n=121, blue regression line): *log*2(*Survivin*) = 8.87772 − 0.26013 ^*^ *log*2(*RPRM*).

We first evaluated available *in silico* data corresponding to an agnostic set of 12 cell-cycle and apoptosis-related genes, two major pathways that have been linked to gastric cancer [26], from the TCGA project [2], using the cBioPortal online platform. Among the genes evaluated by cBioPortal [27, 28], positive correlations were observed between 5 pairs of genes, while an inverse correlation (Pearson’s r = −0.3, Spearman’s ρ = −0.55) was only detected between the expression levels of the Survivin and RPRM transcripts (see online Supplementary Table 2 and Supplementary Figure 1). To validate the latter finding, RNAseq data from 237 selected gastric cancer patients (selection criteria in methods) from the same database were evaluated by linear regression models for Survivin and RPRM. Spearman’s analysis confirmed a significant negative correlation, indicative of mutual exclusion, between Survivin and RPRM transcript expression (ρ = −0.37, p<4.2e-09). To evaluate the linear relationship between Survivin and RPRM, the following linear regression model was proposed:

$${\text{Survivin}}_{i}=\mu +{\text{RPRM}}_{i}+\epsilon_{i}$$

where *Survivin_i_* is the expression level of Survivin for subject “i” (in log 2 scale), *μ* is the overall mean expression of Survivin, *RPRM_i_* is the coefficient associated with the expression level of RPRM (in log 2 scale) for subject “i” and *ϵ_i_* ~ *N* (0, *σ*) is the error term. As shown in Figure 1A, this model was fit to expression data of these two genes.

Next, these samples were segregated according to p53 status into mutated or normal (wild-type) p53 groups, by the following proposed model:

$${\text{Survivin}}_{\mathrm{is}}=\mu +{\text{RPRM}}_{i}+p53_{s}+{\text{RPRM}}_{i}^{*}p53_{s}+\epsilon_{\text{is}}$$

where *Survivin_is_* is the expression level of Survivin (in log 2 scale) for subject “i” with p53 status “s”, *μ* is the overall mean expression of Survivin, *RPRM_i_* is the regression coefficient relative to the RPRM expression levels (in log 2 scale), *p*53_*s*_ is the effect of the p53 subject status, ${\text{RPRM}}_{i}^{*}$ *p*53_*s*_ is the interaction effect, and *ϵ_i_* ~ *N* (0, *σ*) is the error term. The resulting linear model yields statistically significant coefficients for RPRM and their interaction with the p53 status and for the interaction effect: (*RPRMcoefficient* = −0.16, *p* < 0.01) and (RPRM^*^ *p53_wt_coefficient* = −0.10, *p* < 0.05).

These findings imply a highly significant slope decrease of at least 1.58-fold in TCGA cases with wild-type (WT) p53 status when compared to cases with mutated p53 status (Figure 1B). In other words, Survivin down-regulation by RPRM is 1.58 times stronger in WT p53 cases than in mutated p53 cases.

### Expression of Survivin and RPRM in clinical samples

**Figure 2:**
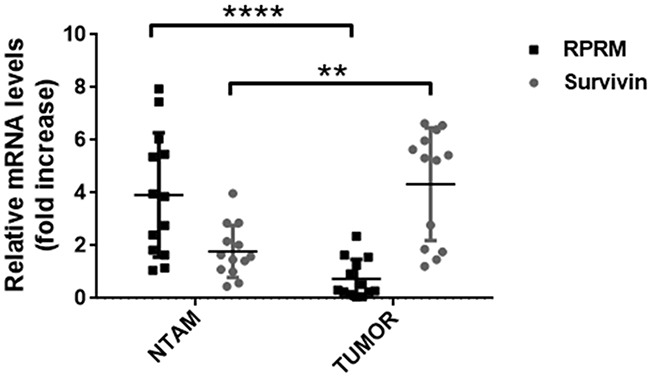
Analysis of Survivin and RPRM mRNA levels in paired tissue samples from primary tumors and non-tumor adjacent mucosa (NTAM) from gastric cancer cases Survivin and RPRM expression levels were evaluated by RT-qPCR in biopsies of tissue samples and normalized to RPS13 mRNA expression levels. Statistically significant differences between RPRM expression in NTAM and tumor samples (^**^p≤ 0.001), as well as for Survivin expression in NTAM and tumor samples (^****^p≤ 0.0001) are indicated (means ± SEM; n = 13 for each sample type).

In order to corroborate our findings from the *in silico* analysis, we evaluated Survivin and RPRM expression in biopsies from NTAM and gastric cancer tumor samples. Analysis by RT-qPCR revealed that Survivin expression was greater in tumors than NTAM (p< 0.001). Conversely, RPRM expression was significantly higher in NTAM than in tumors (p< 0.0001) (Figure 2). These findings were confirmed at the protein level by immunohistochemical analysis of similar samples (see online Supplementary Figure 3).

### Survivin overexpression in cell lines reduces RPRM expression

**Figure 3:**
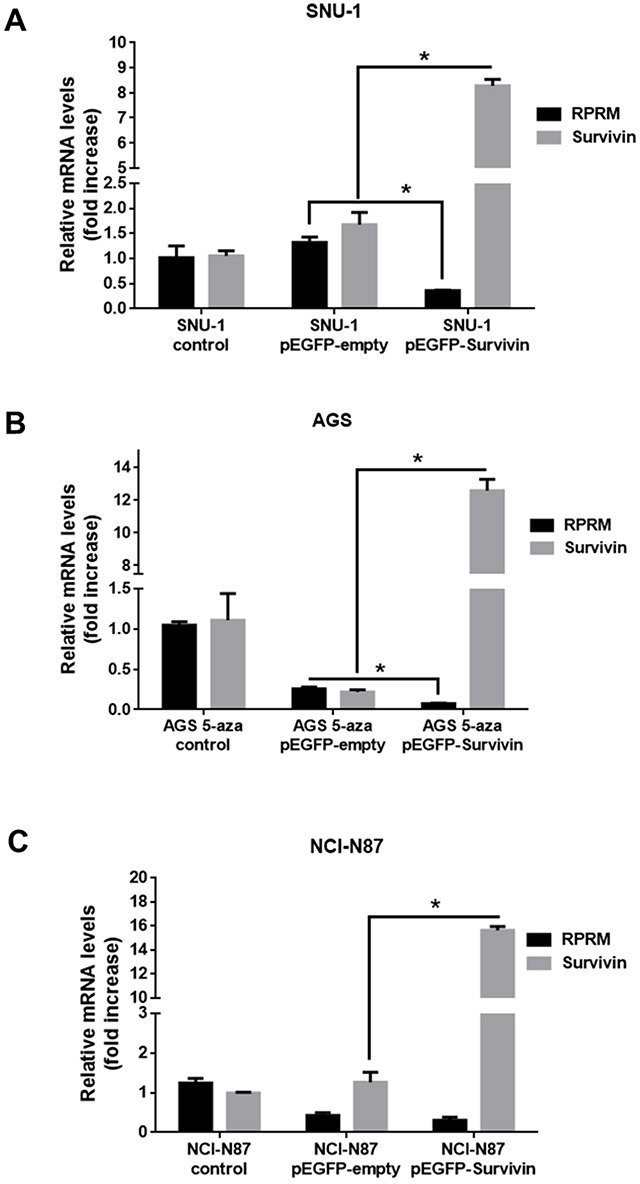
Overexpression of Survivin reduces RPRM mRNA levels in gastric cancer cell lines Survivin and RPRM mRNA expression levels were evaluated by RT-qPCR. Results for **(A)** SNU-1, **(B)** AGS and **(C)** NCI-N87 cells 24 h after transfection with pEGFP-Survivin or pEGFP-empty are shown after normalizing to β-actin mRNA expression levels used as a housekeeping control gene. Note that AGS cells were treated with 5-Aza-2’-deoxycytidine (1 mM) for 24 h prior to transfection. Expression levels in non-transfected cells were used to standardize each experiment (control). Statistically significant differences compared to cells transfected with pEGFP-Survivin or empty vector are shown (means ± SEM; n = 4; Mann-Whitney test; ^*^p< 0.05).

To determine the effects of Survivin on RPRM expression, three gastric cancer cell lines were transfected with a plasmid encoding Survivin. As expected, for all cell lines a significant increase in Survivin transcript (p< 0.05) and protein expression levels was detectable 24h post-transfection (Figure 3). Alternatively, RPRM mRNA expression levels in SNU-1 cells 24h after transfection with pEGFP-Survivin were considerably reduced as compared with the pEGFP-empty transfected cells (p< 0.05) (Figure 3A). Additionally, to restore RPRM in AGS cells, where expression is suppressed by promoter methylation, we treated the cells with 5′-Aza, an inhibitor of DNA methyl transferases (see online Supplementary Figure 4). Also in this case, we observed that overexpression of Survivin using the pEGFP-Survivin plasmid reduced RPRM expression significantly (p< 0.05) in comparison to the pEGFP-empty cells (Figure 3B). Conversely, there were no statistically significant differences in RPRM transcript expression between NCI-N87 cells transfected with the plasmid encoding Survivin or empty plasmid (Figure 3C). Also, we transfected HEK-293T cells with pEGFP-Survivin as a normal control. Surprisingly, relative RPRM mRNA levels increased rather than decreased upon Survivin expression in these cells (see Supplementary Figure 5A).

### RPRM overexpression reduces Survivin expression

**Figure 4:**
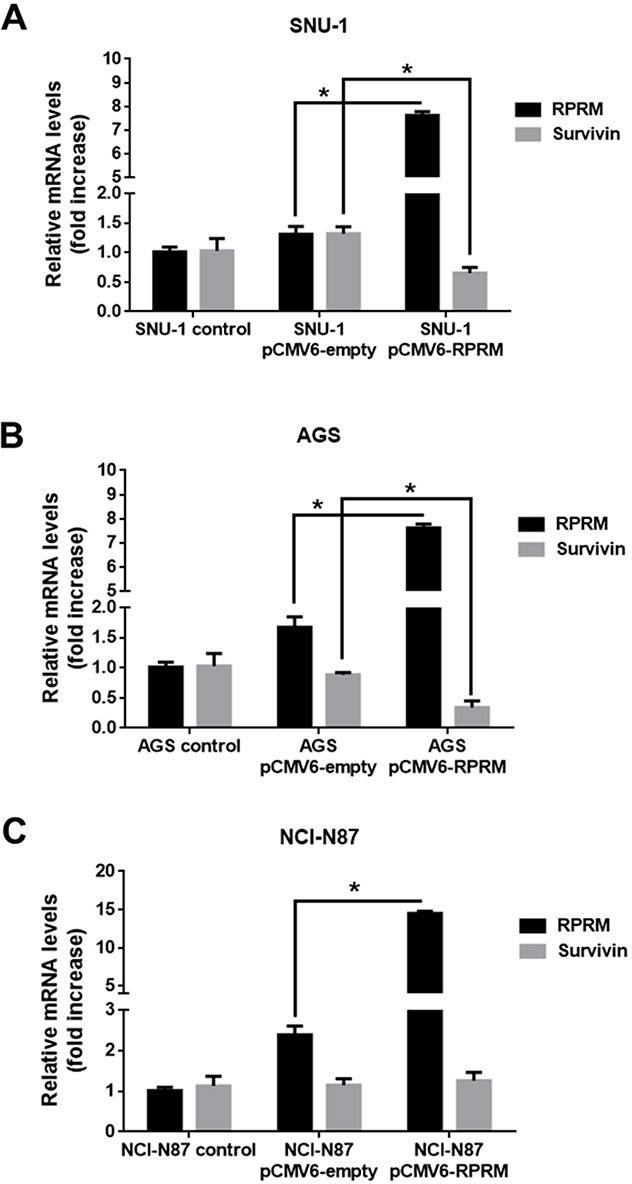

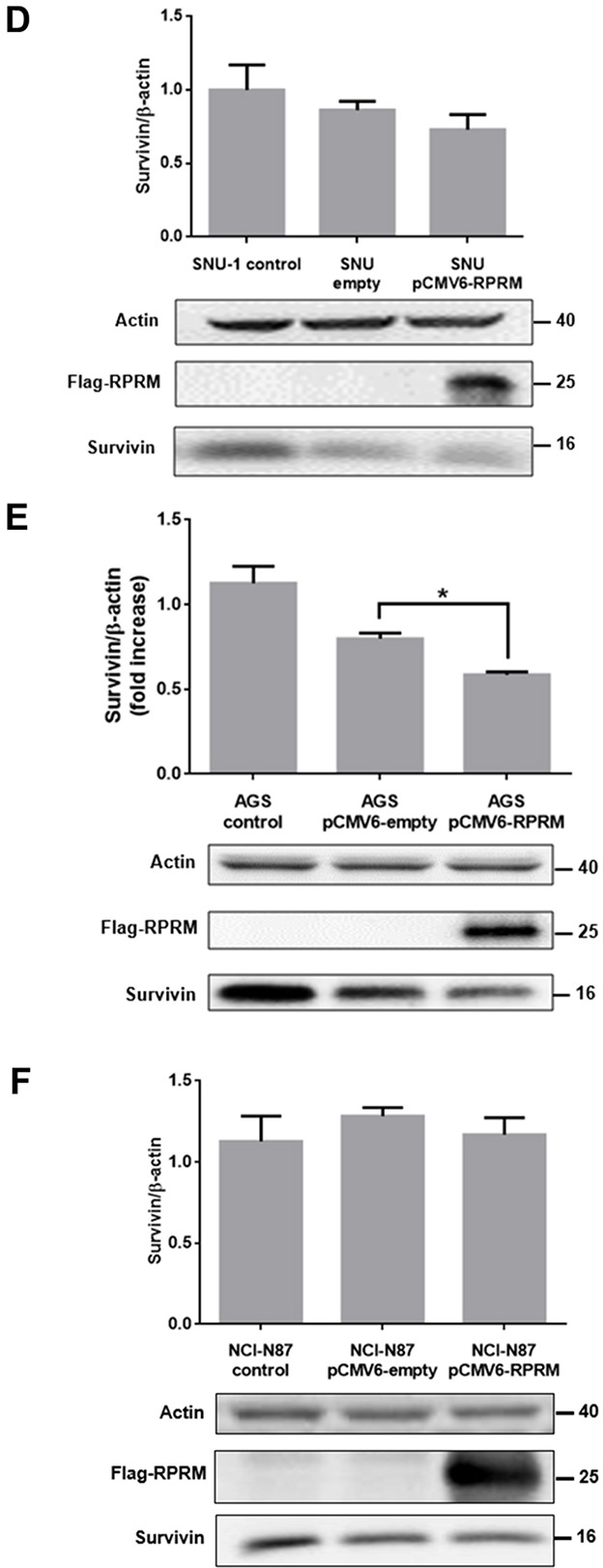
Overexpression of RPRM reduces Survivin levels in gastric cancer cell lines Survivin and RPRM expression levels were evaluated by RT-qPCR in **(A)** SNU-1, **(B)** AGS and **(C)** NCI-N87 cells 24 h after transfection with either pCMV6-empty or pCMV6-RPRM. Values were normalized to β-actin mRNA expression levels used as a housekeeping gene. Expression levels in non-transfected cells were used to standardize each experiment (control). Protein levels assessed by Western blot analysis for **(D)** SNU-1, **(E)** AGS and **(F)** NCI-N87 cells 24 h after transfection with empty vector or pCMV6-RPRM normalized to β-actin are shown. Statistically significant differences compared to cells transfected with pCMV6-RPRM or empty plasmid are indicated (means ± SEM; n = 4; Mann-Whitney test; ^*^p≤ 0.05).

To confirm the mutual exclusivity hypothesis between Survivin and RPRM, we also increased RPRM expression levels by transiently transfecting with pCMV6-RPRM. As expected, for all cell lines a significant increase in RPRM transcript (p< 0.05) and protein expression levels (p< 0.05) were detected 24h post-transfection (Figure 4). Survivin mRNA expression levels significantly diminished in SNU-1 cells transfected with plasmid encoding RPRM as compared with the empty plasmid (p< 0.05) (Figure 4A). Also, RPRM overexpression in AGS cells significantly reduced Survivin expression levels (p< 0.05) as compared with pCMV6-empty vector (Figure 4B). Conversely, there were no statistically significant differences in Survivin mRNA expression levels in transfected NCI-N87 cells overexpressing or not RPRM (Figure 4C). On the other hand, when HEK-293T cells were transfected with pCMV6-RPRM as a normal control, Survivin mRNA levels remained essentially unchanged (see online Supplementary Figure 5B).

To investigate whether RPRM overexpression altered Survivin protein levels in the cells, we analyzed extracts from pCMV6-RPRM and pCMV6-empty transfected cells by western blotting. The analysis revealed a noticeable, but statistically insignificant decrease in Survivin protein levels in SNU-1 cells (Figure 4D). Alternatively, in AGS cells, a significant decrease in Survivin expression levels was observed following transfection within pCMV6-RPRM as compared with the control pCMV6-empty vector transfected cells (p<0.05) (Figure 4E). Conversely, when we overexpressed RPRM in NCI-N87 cells, no statistically significant differences in Survivin protein levels were observed as compared with empty plasmid control cells (Figure 4F). On the other hand, when HEK-293T cells (normal control) were transfected with pCMV6-RPRM, Survivin protein levels increased (see online Supplementary Figure 5C).

### Clinical significance of Survivin and RPRM expression in gastric cancer

**Figure 5:**
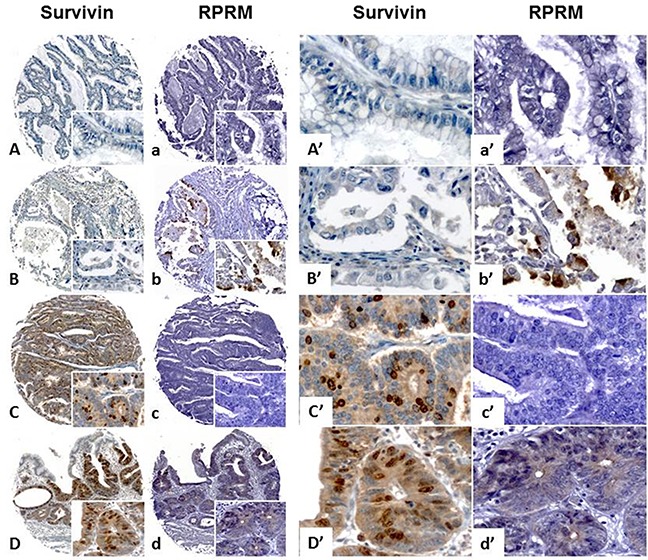
Analysis of Survivin and RPRM protein expression levels in a tissue microarray containing gastric cancer cases Survivin and RPRM expression levels evaluated by immunohistochemistry on a tissue microarray of gastric cancer cases for clinicopathological correlations. Survivin and RPRM presence in cells is revealed as brown staining. All samples were counterstained with hematoxylin (blue nuclei). The original magnification for images **(A-D)** and **(a-d)** was 100x (image at bottom right) and 400x for images **(A’-D’)** and **(a’-d’)**. The scale bar setting for all images was 100 μm. Survivin and RPRM protein levels were semi-quantified using the median expression method (see methods for details) in TMA sections. Two cases did not show mutual Survivin and RPRM exclusion, being negative (A-a) or positive (D-d) for both genes. B-b and C-c show mutual exclusion for Survivin and RPRM, being either Survivin(-)/RPRM(+) or Survivin(+)/RPRM(−).

**Figure 6:**
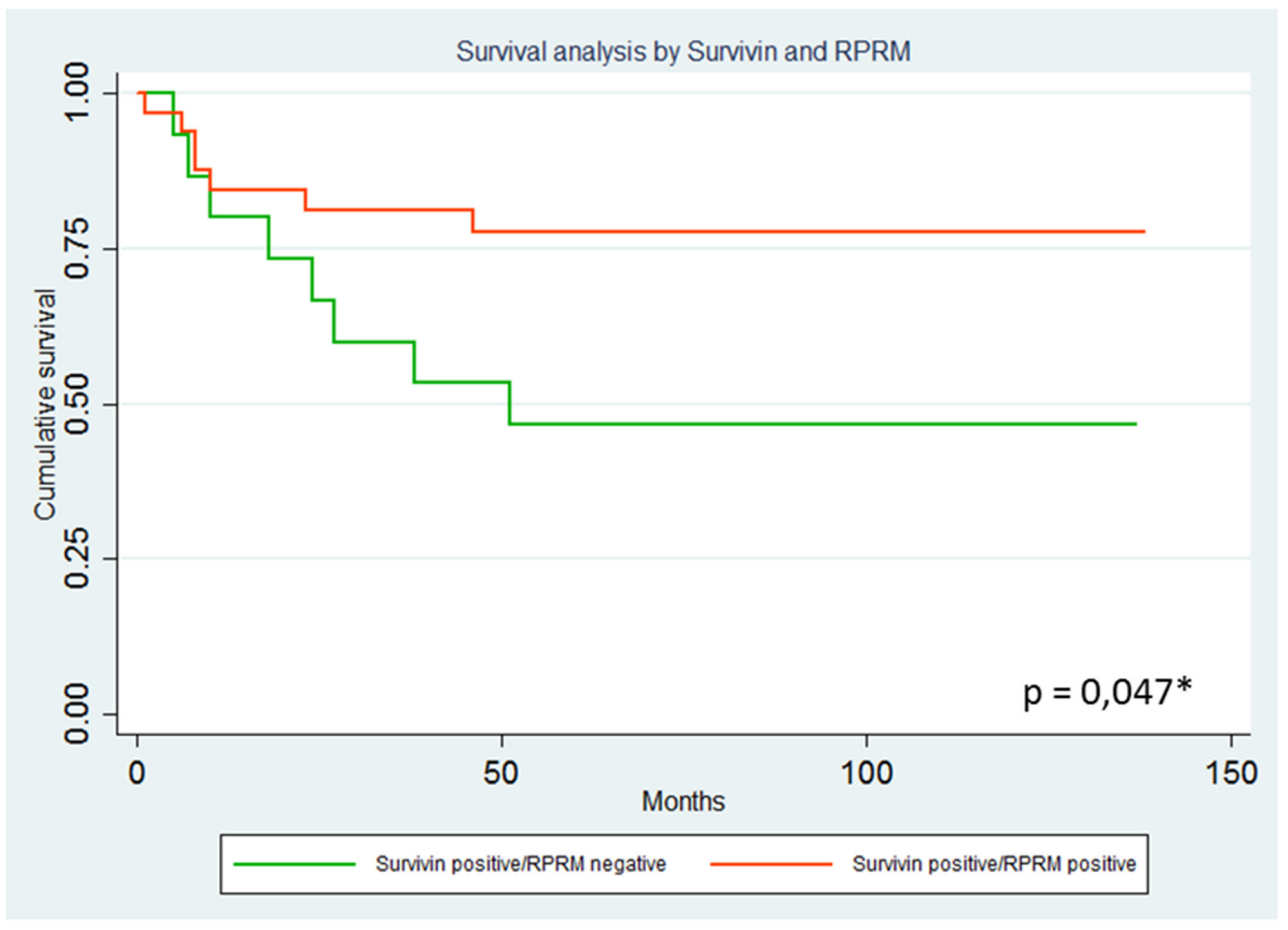
Analysis of overall survival curves among Survivin positive cases according to RPRM protein expression levels Results are shown comparing 21 Survivin positive/RPRM positive and 17 Survivin positive/RPRM negative cases. Overall survival analysis by Kaplan-Meier estimation considering Survivin and RPRM protein expression after correction for age and sex. A worse prognosis among Survivin-positive patients is observed in RPRM negative cases (p=0.047, Long Rank Test) (HR=0.32; 95%IC: 0.11-0.97; p=0.044).

To explore the possible clinical significance of the mutual exclusion between Survivin and RPRM, a TMA containing 114 cases was immunohistochemically evaluated for expression of both proteins and correlated with clinicopathological variables, as well as overall survival. As shown in Figure 5, among the 107 available cases, Survivin protein expression was found in 47.6% (57/107) of cases (>10% staining). On the other hand, RPRM protein was expressed in 38.3% (41/107) of cases (>20% staining). To evaluate the clinicopathological significance of these findings, cases adjusted by age and sex were compared. Overall, no associations were found (data not shown). However, a worse prognosis among Survivin-positive / RPRM-negative cases was detected (p=0.047, Long Rank Test) (HR=0.32; 95%IC: 0.11-0.97; p=0.044) (Figure 6).

## DISCUSSION

Deregulation of p53-mediated cellular processes, such as apoptosis and cell cycle control signaling pathways, have been associated with the progression of gastric cancer [12, 26, 29, 30]. In the current study, the TCGA database was interrogated to identify deregulated p53-associated cell cycle and apoptosis genes in gastric cancer. In doing so, the pair Survivin and RPRM stood out because a highly significant negative correlation was detected between the two genes at the mRNA level. In functional terms, given that both genes regulate cell cycle progression in opposing ways at the G2/M transition [24, 31], this negative correlation was considered potentially relevant to gastric cancer development. Thus, we then validated these findings at the RNA and protein levels in clinical samples from gastric cancer patients. We also corroborated these findings in gastric cell lines by showing that Survivin overexpression suppressed RPRM and conversely RPRM overexpression reduced Survivin levels. Of note, we observed significant down-regulation only in cells expressing wild-type p53, but not in cells with mutated p53. These results are consistent with the initial *in silico* analysis of TCGA data, where the negative correlation between Survivin and RPRM expression was notably accentuated in patients with wild-type p53 protein. The clinical significance of these findings was underscored by showing that survival rates of Survivin-positive gastric cancer patients was significantly reduced when RPRM expression is lost.

Survivin is poorly expressed in the G1 phase, increases by 6-fold in the S phase and by more than 40-fold in G2/M phase of the cell cycle (reviewed in Jaiswal et al., [32]. Thus, the presence of Survivin favors G2/M progression, cell survival and proliferation, all functions diametrically opposed to the documented role of RPRM. Given that p53 favors RPRM expression, the fact that Survivin reportedly also reduces p53 expression [4, 23] could explain how Survivin controls RPRM in a p53-dependent manner, as indicated by our results (Figure 3).

PI3K/Akt activity is known to favor the expression of Survivin in VEGF-stimulated endothelial cells and contribute thereby to cell survival and angiogenesis [33]. More recently, Survivin expression in cancer cells was linked to activation of the PI3K/Akt/beta-catenin/Tcf-Lef pathway, to increase VEGF expression by these cells and promote angiogenesis. Therefore, in doing so, Survivin expression in cancer cells participates in a positive feedback amplification loop, that augments PI3K/Akt activity, promotes VEGF liberation, angiogenesis and tumorigenesis [34–36]. Given the importance of p53 loss in gastric cancer progression, the fact that PI3K/Akt activity directly favors p53 down-regulation by promoting Mdm2–mediated proteasomal degradation [37] posits enhanced Survivin expression as a potentially key determinant in gastric cancer progression by generating a feedback amplification loop that would not only promote PI3K/Akt activity, but also favor the loss of p53 and RPRM. While highly intriguing, further studies are required to substantiate this possibility.

In contrast to Survivin, little information is available concerning RPRM regulation and activity. RPRM induces cell cycle arrest at the G2/M phase and RPRM expression is enhanced by p53 upon exposure to DNA damage [16]. Functional assays, such as colony formation and anchorage-independent growth assays, point towards a putative tumor suppressor role for RPRM in gastric cancer cells [20]. In addition, epigenetic silencing of the RPRM gene by promoter methylation is associated with loss of RPRM expression in gastric cancer cells [38]. Accordingly, RPRM is often lost in invasive stages of gastric cancer [20]. For these reasons, in our experiments, we also used the demethylating agent 5′-azacytidine to restore RPRM expression in AGS cells. When we transfected these cells with pEGFP-Survivin a significant decrease in RPRM was observed (Figure 3B). Unfortunately, however, the transfection with empty-vector also had a substantial effect on RPRM expression. This represents a limitation to our study, perhaps reflecting the poor specificity of the demethylating agent. Recently, reprogrammed re-expression of RPRM by CRISPR/dCas9 system was reported to induce beneficial effects in the AGS cell line [21]. This could represent a useful tool to specifically restore RPRM expression in these cells in future studies. Nonetheless, our results in SNU-1 cells (Figure 4A) indicate that overexpression of RPRM sufficed to reduce Survivin expression, and that p53 was required because such RPRM-induced changes were not seen in p53-deficient NCI-N87 cells (see Figure 3C). In gastric cell lines, we also corroborated these findings by showing that Survivin overexpression suppressed RPRM and conversely RPRM overexpression reduced Survivin. Of note, we observed significant down-regulation only in cells expressing wild-type p53, but not in cells with mutated p53 (see results with NCI-N87 cells, Figures 3C and 4C). These results are consistent with the initial results obtained by *in silico* analysis of the TCGA data, where the inverse correlation between Survivin and RPRM expression was highly accentuated in patients with wild-type p53 protein (Figure 1).

These observations mentioned above indicate that loss of RPRM by promoter region methylation could favor disease progression by augmenting Survivin. Accordingly, our results concerning Survivin and RPRM protein expression in a large set of clinical samples validate these *in vitro* findings by revealing substantially diminished 5-year survival among the paired Survivin-positive/RPRM-negative gastric cancer cases (Figure 6). Taken together, these findings not only confirm at the clinical level the inverse correlation between Survivin and RPRM expression, but also identify a protective effect and enhanced survival for those Survivin positive-gastric cancer cases in which RPRM is co-expressed.

Clearly, further research will be required to shed light on the detailed molecular mechanisms and signaling pathways linking Survivin and RPRM regulation to one another, as well as the therapeutic potential that may be derived from such insight.

## MATERIALS AND METHODS

### *In silico* analysis of TCGA RNAseq data

An initial exploratory analysis was performed on data from the stomach adenocarcinoma (STAD) TCGA [2] using the online cBioPortal for Cancer Genomics platform (http://www.cbioportal.org) [27, 39]. A total of 258 cases were selected, corresponding to the “All Complete Tumors” option of the Stomach Adenocarcinoma “TCGA, Nature” tab from cBioPortal (see online Supplementary Table 1). Given our interest in analyzing the correlation between cell cycle and apoptosis, Pearson’s and Spearman’s correlation values were retrieved using the “Co-expression” tab from the cBioPortal for an agnostic set of twelve p53-dependent apoptosis- and cell cycle- related genes (see online Supplementary Table 2 and Supplementary Figure 1). Subsequently, RNAseqV2 data from all tumor samples available from the STAD TCGA were downloaded using the DownloadRNASeqData function included in Module_A from TCGA-Assembler [40], on R statistical programming language. Data were processed using the ProcessRNASeqData function included in Module_B from TCGA-Assembler (see online Supplementary Figure 2). Of these, 237 cases met the following criteria: i) Survivin and RPRM expression were both greater than 0, and ii) p53 mutational status information (mutated p53 n=116 and wild-type p53 n=121) was available on cBioPortal (see online Supplementary Table 3). The p53 status was manually retrieved from cBioPortal and cross-referenced to the RNAseq expression matrix using the unique TCGA identifiers (barcode) for each case. For R code, see Supplementary Material.

### Clinical samples

Thirteen de-identified matched tumor and NTAM fresh-frozen human tissue samples obtained from upper gastrointestinal endoscopic procedures at the Instituto Chileno-Japones de Enfermedades Digestivas – Hospital Clinico San Borja-Arriaran (ICHJED-HCSBA) were evaluated for Survivin and RPRM expression by quantitative reverse transcription PCR (RT-qPCR). Eighteen de-identified matched tumor and NTAM formalin-fixed and paraffin-embedded samples from the pathology archives at the ICHJED-HCSBA were selected for the immunohistochemical evaluation of Survivin and RPRM expression. A previously reported TMA cohort of 114 consecutive gastric cancer cases [20, 41], stratified according to the WHO Classification of Gastric Cancer, the Japanese Research Society for Gastric Cancer recommendations and the AJCC gastric cancer staging system [18, 42, 43] with a 12 year follow-up [44], was evaluated for the clinical significance of the mutual exclusion between Survivin and RPRM. All cases selected from the pathology archives of the ICHJED-HCSBA had undergone subtotal or total gastrectomy as the only treatment [44]. Clinico-pathological correlations, but not follow-up survival, have been reported for RPRM in these cases [20]. Written informed consent was obtained from each participant and protocols were approved by the Ethics Committee of the Pontificia Universidad Católica de Chile and ICHJED-HCSBA.

### Quantitative reverse transcription polymerase chain reaction (RT-qPCR)

Total RNA was isolated with TriZOL™ (Life Technologies, Carlsbad, CA, US) according to manufacturer’s instructions and then used as a template to synthesize first-strand complementary DNAs (cDNA) by reverse-transcription PCR with Oligo dT primers (Promega, Madison, WI, US) and Moloney Murine Leukemia Virus reverse transcriptase (Promega, Madison, WI, US). The cDNA was amplified by RT-qPCR using 5x HOT FIREPol Evagreen® qPCR Mix Plus (Solis Biodyne, Riia, Tartu, Estonia) according to manufacturer’s instructions with the following primer pairs: for Survivin, sense primer 5′-CTGGCAGCCCTTTCTCAAGGA-3′ and anti-sense primer 5′-GCAACCGGACGAATGCTTTT-3′; for RPRM, sense primer 5′-GAGCGTAGCCTGTACATAA TGC-3′ and anti-sense primer 5′-CCTTCACGAGGAAG TTGATCAT-3′; for beta-actin, sense primer 5′-AAAT CGTGCGTGACATTAAGC-3′ and anti-sense primer 5′-CCGATCCACACGGAGTACTT-3′; for RPS13, sense primer 5′-CTCTCCTTTCGTTGCCTGAT-3′ and anti-sense primer 5′-TGAAGGAGTAAGGCCCTTCT-3′. All reaction products were analyzed after 40-45 amplification cycles with the following thermal profile: activation 1s at 25°C and 10min at 95°C, denaturation 15s at 95°C, 30 s at 98°C, annealing 18s at 72°C and extension 15s at 95°C, 1s at 25°C, 15s at 70°C and 1s at 95°C. Relative fold-increases in gene expression levels were calculated using the MIQE (minimum information for publication of quantitative real-time PCR experiments) guidelines [45]. Survivin and RPRM expression was normalized to transcript levels of the RPS13 housekeeping gene for biopsy specimens and then expressed relative to values obtained for NTAM samples. Alternatively, values obtained for cell lines were standardized to beta-actin as a housekeeping gene and then expressed relative to expression obtained for wild-type cell extracts (control) in each transfection condition (value = 1).

### Immunohistochemistry

Survivin and RPRM protein expression levels were evaluated using formalin-fixed and paraffin-embedded tissue samples. Survivin and RPRM were detected using the Vectastain Elite Kit R.T.U (Vector Laboratories, Ingold Road, CA, US), according to the manufacturer’s instructions, with a polyclonal anti-Survivin antibody (R&D Systems, Minneapolis, MN, USA) and a polyclonal anti-RPRM antibody (Sigma, St Louis, MO, US) [6, 20]. Results of TMA immunostaining were considered positive based on the median of protein expression. In the case of RPRM, the median of cytoplasmic expression in epithelial cells was 20%. In the case of Survivin median expression of nuclear staining was 10%. Stained tissue sections were evaluated by two independent pathologists (GCA and AHC) who were unaware of the clinical data. Supplementary results of whole block immunostaining for NTAM and tumor tissues were evaluated using Quick Score (Q Score) analysis as described [46].

### Cell lines and culture conditions

The gastric cancer cell lines AGS (ATCC CRL 1793), SNU-1 (ATCC CRL 5971) and NCI-N87 (ATCC CRL 5822) were cultured in Roswell Park Memorial Institute 1640 medium (Gibco BRL, Carlsbad, CA, USA). The human embryonic kidney cell line HEK293T (ATCC CRL 3216) was cultured in Dulbecco’s modified Eagle medium (Gibco BRL, Carlsbad, CA, USA). According to data sheet information provided by ATCC, all cells used here have the p53 wild-type gene, except for NCI-N87 cells, where the p53 gene is mutated. In all cases, culture media were supplemented with 10% fetal bovine serum (Biological Industries, Sebethe Drive, Cromwell, CT, US) and antibiotics (10,000 U/ml penicillin and 10 mg/ml streptomycin), and cells were cultured at 37°C in a humidified atmosphere containing 5% CO2.

### Transfections

SNU-1, AGS, NCI-N87 and HEK293T cells (3×10-5 in each case) were transfected in 6-well plates with pEGFP-empty, pEGFP-Survivin or pCMV6-empty, pCMV6-RPRM (tagged with FLAG)[6, 20] using the ViaFect™ Transfection Reagent (Promega, Madison, WI, US) according to manufacturer’s instructions. Prior to transfection with pEGFP-empty and pEGFP-Survivin, AGS cells were treated with the demethylating agent 5′-Aza-2′-deoxycytidine (5′-Aza) at a concentration of 1 μM for 24 h in order to revert epigenetic RPRM silencing via promoter methylation [20].

### Western blot analysis

Cells were harvested and whole-cell lysates were prepared by sonicating in buffer (NP-40 and SDS 10%), supplemented with a protease and phosphatase inhibitor cocktail (Sigma Aldrich, St Louis, MO, US). Protein concentrations were determined using the BCA Protein Assay reagent (Pierce, Thermo Scientific, Logan, Utah, USA) according to the manufacturer's instructions. Equal amounts of total cellular protein (50ug/lane) were separated by sodium dodecyl sulfate–polyacrylamide gel electrophoresis on 12% acrylamide minigels (Bio-Rad Laboratories, Hercules, CA, US) and transferred to nitrocellulose membranes (Amersham™Protran™0.45μm, Life Science, Sigma, St Louis, MO, US). The membranes were blocked as described previously [34] and then probed with rabbit anti-beta-actin polyclonal antibody (1:5000, A5060, Sigma, St Louis, MO, US) as a loading control, mouse anti-FLAG monoclonal antibody (1:2000, F3165, Sigma, St Louis, MO, US) or rabbit anti-human Survivin polyclonal antibody (1:3000, AF886, R&D Systems, Minneapolis, MN, USA). Horseradish peroxidase-conjugated anti-rabbit (1:3000, AP132P, Millipore, Merck, Darmstadt, Germany) or anti-mouse secondary antibodies (1:3000, 1706516, Bio-Rad Laboratories, Hercules, CA, US) were used to detect primary antibodies with the EZ-ECL system (Biological Industries, Sebethe Drive, Cromwell, CT, US) according to the manufacturer's protocol. Western blots were used to quantify protein levels by scanning densitometry as described previously [28].

### Statistical analysis

Downloaded RNAseqV2 data were statistically evaluated by Spearman correlation and linear regression analysis using the R statistical programming environment. Data from cell lines and clinical samples were compared pairwise between cells transfected with empty plasmid or plasmid encoding the indicated insert sequence, and NTAM or tumor samples, respectively. Values were analyzed using the Mann-Whitney test and GraphPad Prism software (version 6.0, San Diego, CA, US). All data were obtained from 3 or more independent experiments and were expressed as mean ± SEM. Clinicopathological and overall survival were determined by the Kaplan-Meier test. Differences between survival rates were assessed using the Long-Rank test. All calculations were performed by STATA v14,0. A p value of less than 0.05 was considered statistically significant [47].

## SUPPLEMENTARY MATERIALS FIGURES AND TABLES











## Abbreviations

TCGA: The Cancer Genome Atlas
EBV: Epstein-Barr virus
TP53: tumor protein p53
BIRC5: Survivin
RPRM: Reprimo
IAP: Inhibitor-of-apoptosis protein
STAD: Stomach adenocarcinoma
RT-qPCR: Quantitative reverse transcription polymerase chain reaction
NTAM: non-tumor adjacent mucosa
