# Supplementary material for: Inverse expression of survivin and reprimo correlates with poor patient prognosis in gastric cancer
Source: Oncotarget. 2018 Feb 5;9(16):12853–67. doi: 10.18632/oncotarget.24402 (PMC5849179; doi:10.18632/oncotarget.24402)
Supplement: Supplementary file 1 [file oncotarget-09-12853-s001.pdf]

Inverse expression of survivin and reprimo correlates with poor patient prognosis in gastric cancer

SUPPLEMENTARY MATERIALS

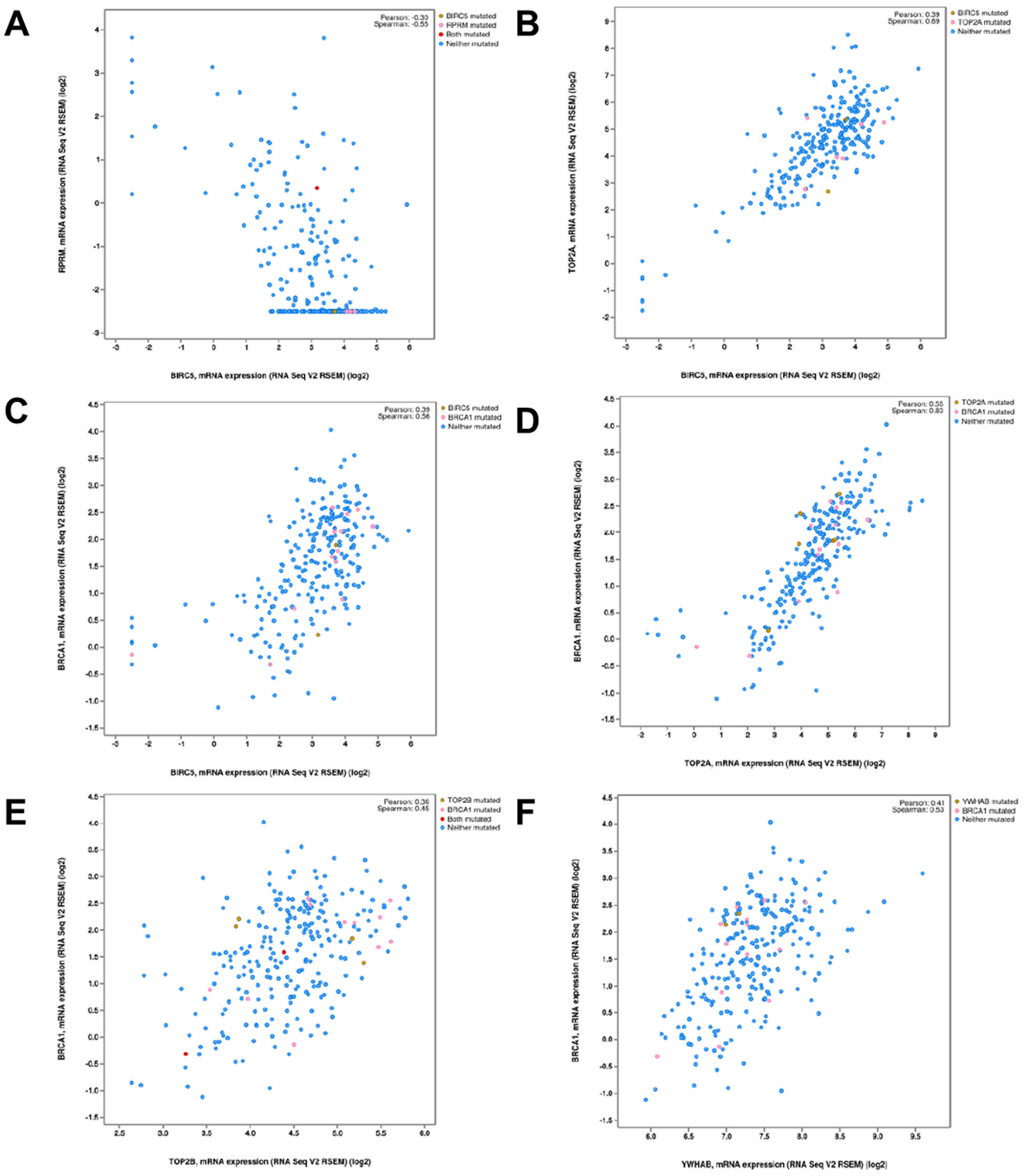

Supplementary Figure 1: Correlation analysis between apoptosis and cell cycle related genes data from the TCGA (cBioPortal). Six pairs of genes from an agnostic set of 12 cell-cycle and apoptosis-related genes showed Pearson's and Spearman's correlation ≤|0.3|≤ (lower than or equal to -0.3; or higher than or equal to 0.3): **(A)** RPRM (Y-axis) and Survivin (X-axis) (P: -0.3 and Sp: -0.55); **(B)** TOP2A (Y-axis) and Survivin (X-axis) (P: 0.39 and Sp: 0.69), **(C)** BRCA1 (Y-axis) and Survivin (X-axis) (P: 0.39 and Sp: 0.56), **(D)** BRCA1 (Y-axis) and TOP2A (X-axis) (P: 0.55 and Sp: 0.83), **(E)** BRCA1 (Y-axis) and TOP2B (X-axis) (P: 0.36 and Sp: 0.45), **(F)** BRCA1 (Y-axis) and YWHAB (X-axis) (P: 0.41 and Sp: 0.53) (n=258). P = Pearson's r, Sp = Spearman's ρ.

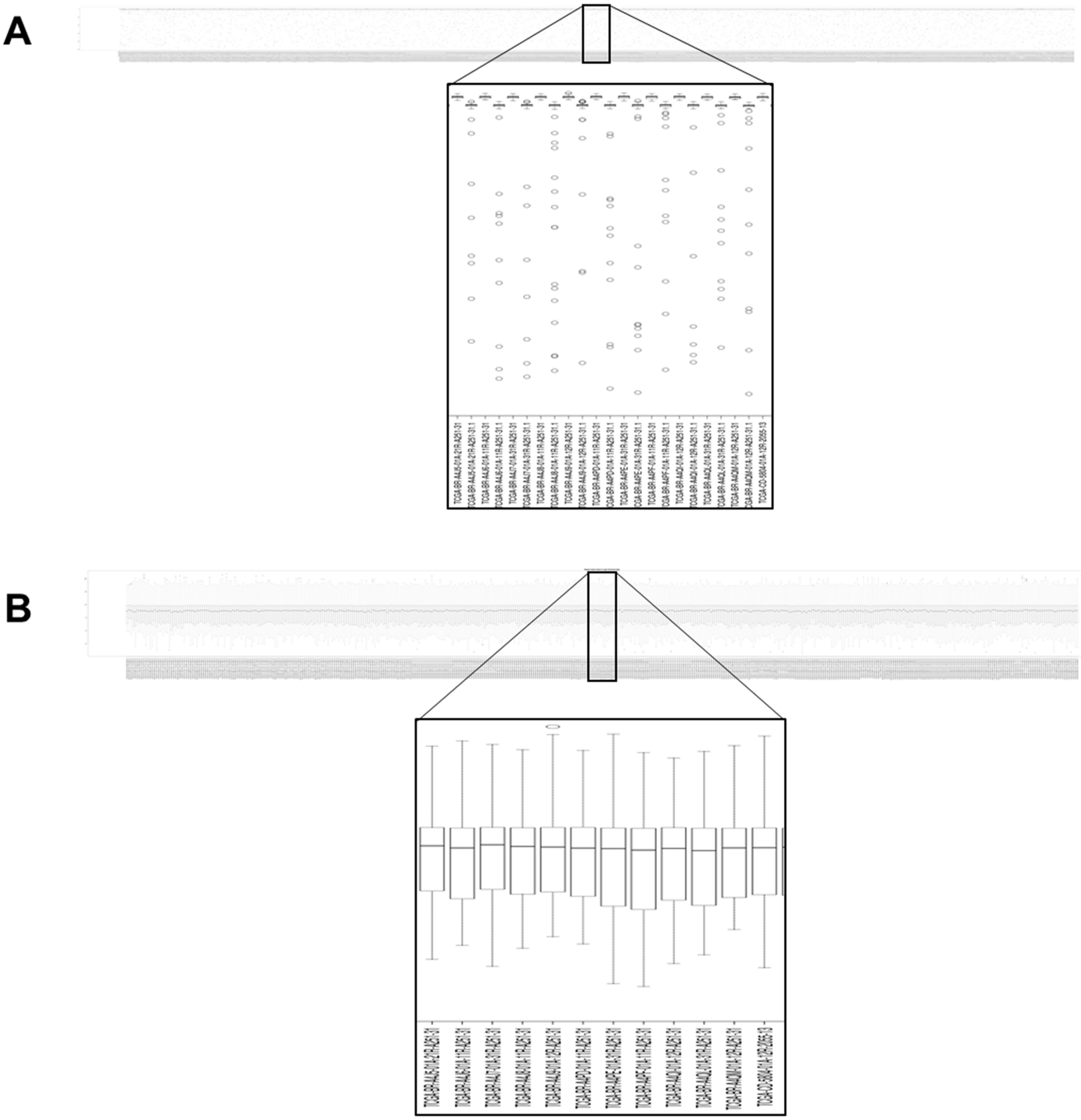

Supplementary Figure 2: Normalization of Stomach Adenocarcinoma TCGA RNAseqV2 data. **(A)** Boxplots showing the distribution of raw RNAseqV2 data from the stomach adenocarcinoma TCGA study downloaded from the TCGA repository using the DownloadRNASeqData function from TCGA-Assmbler's Module_A (n=450). Each boxplot represents a different sample. **(B)** Boxplots showing the distribution of normalized RNAseqV2 data from the stomach adenocarcinoma TCGA study. Raw data from A were processed and normalized using the ProcessRNASeqData function from TCGA-Assembler's Module_B. Each boxplot represents a different sample.

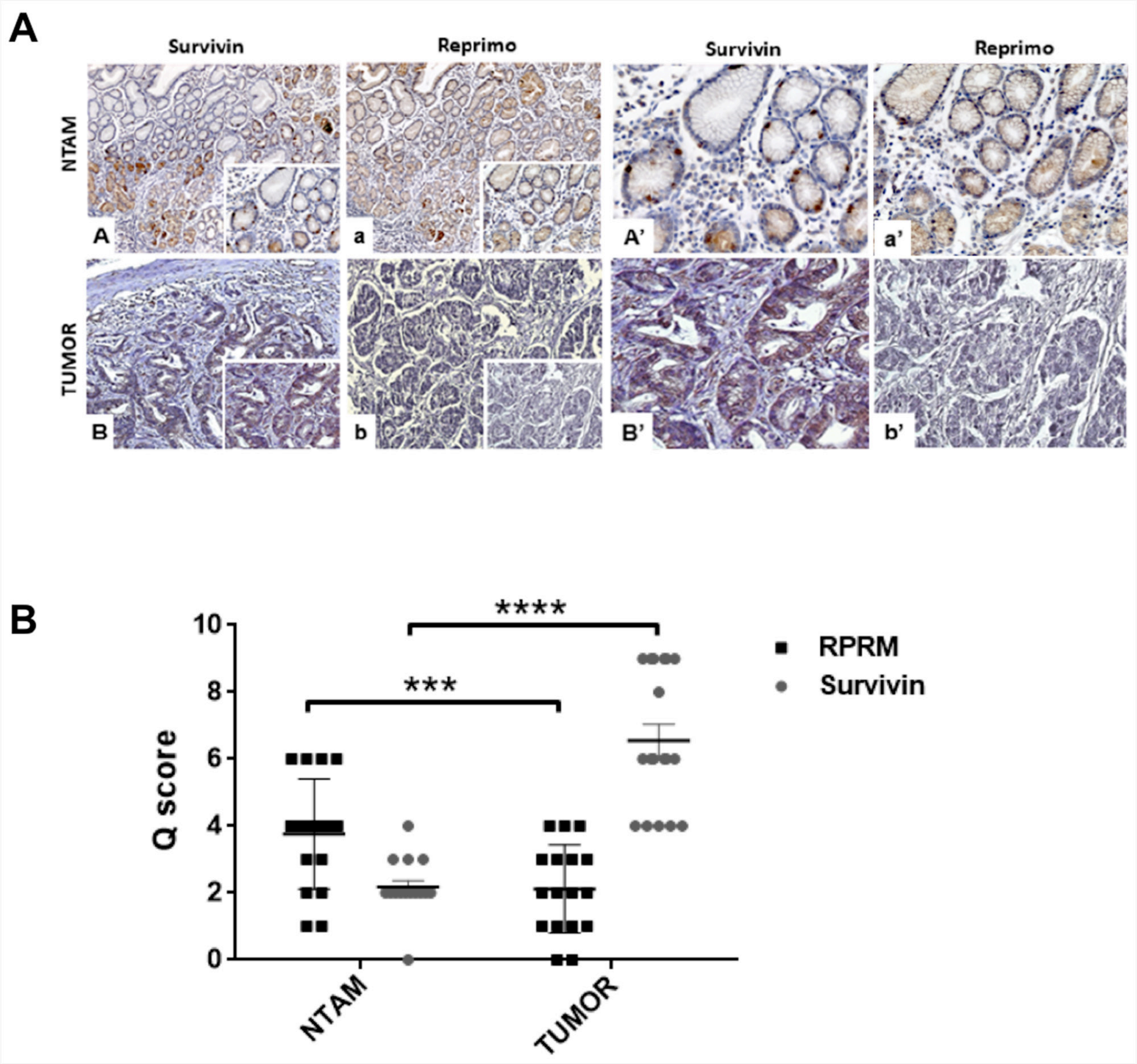

Supplementary Figure 3: Immunohistochemical analysis of Survivin and RPRM in whole tissue samples from human gastric cancer tissues (matched normal adjacent mucosa and tumor samples). Survivin and RPRM transcript expression levels were evaluated by immunohistochemistry in formalin-fixed and paraffin-embedded gastric cancer tissue samples. Survivin and RPRM staining in cells are visible as brown staining. All samples were counterstained with hematoxylin (blue nuclei). Representative immunostained samples for Survivin and RPRM in NTAM and tumor samples are shown in **(A)**. The original magnification for images **(A-B)** and **(a-b)** was 200x (image at bottom right) and 400x for images **(A'-B')** and **(a'-b')**. The scale bar setting for all images was 100 µm. **(B)** Survivin and RPRM protein levels were evaluated using the Q Score method (see methods for details). Results for Survivin and RPRM expression in NTAM and tumor samples are shown (means ± SEM; n = 18 ;Mann-Whitney test; $^{***}p= 0.0004$, $^{****}p< 0.0001$, respectively).

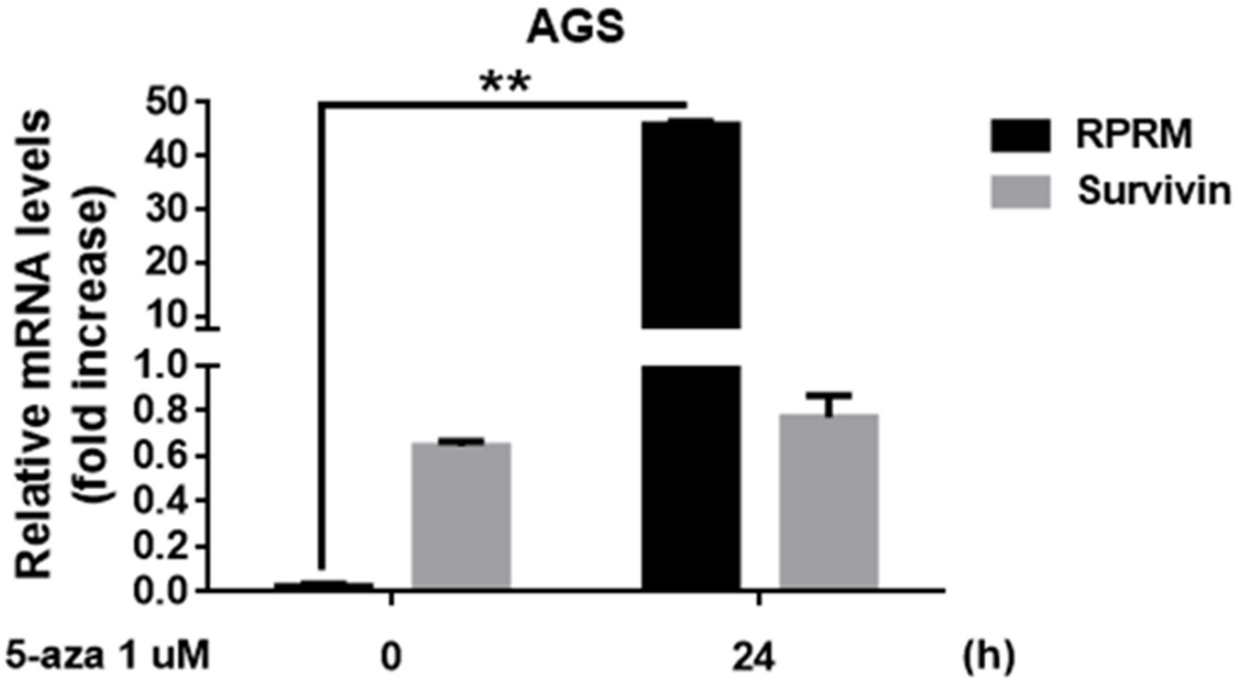

Supplementary Figure 4: Survivin and RPRM expression in AGS cells previously treated with 5-Aza-2'-deoxycytidine. Survivin and RPRM transcript expression levels were evaluated by Real-Time PCR in wild-type AGS cells and AGS cells previously treated with 5-Aza-2'-deoxycytidine 1 mM for 24 h. Values were normalized to β-actin mRNA expression. Statistically significant differences between wild-type cells and treated AGS cells are shown (means ± SEM; n = 4; Mann-Whitney test; $^{**}p < 0.001$).

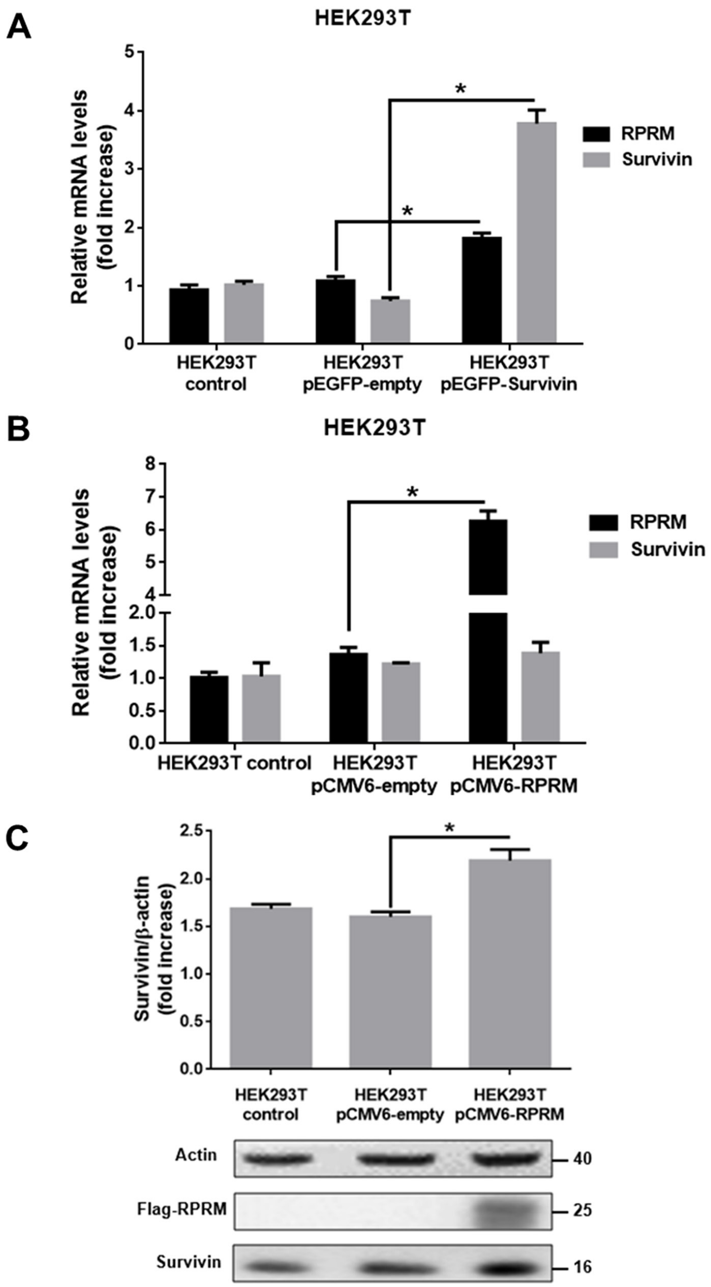

Supplementary Figure 5: Survivin and RPRM expression in HEK293T cell line. Survivin and RPRM expression levels were evaluated in HEK293T cells by Real-Time PCR. **(A)** 24 h after transfection with pEGFP-empty or pEGFP-Survivin. **(B)** 48 h after transfection with pCMV6-empty and pCMV6-RPRM. All values were normalized to β-actin mRNA expression. Non-transfected cells were used to standardize values for each condition (control). **(C)** protein levels were determined by Western blot analysis of extracts from cells 24 h after transfection with either pCMV6-RPRM or empty vector. Values were normalized to β-actin protein levels. Statistically significant differences comparing cells transfected either with vector plus coding sequence or with empty vector are shown (means ± SEM; n = 4; Mann-Whitney test; $^{*}p \leq 0.05$).

Supplementary Table 1: List of 258 cases selected by the Stomach Adenocarcinoma “TCGA, Nature” tab from cBioPortal for exploratory correlation analysis.

See Supplementary File 1

Supplementary Table 2: Exploratory correlation analysis between pairs of apoptosis and cell cycle genes from the Stomach Adenocarcinoma “TCGA, Nature” tab (cBioPortal). An agnostic set of eleven p53-dependent apoptosis- and cell cycle-related genes (Survivin, SHISA5, IGFBP3, BAX, FAS, RPRM, TOP2A, TOP2B, YWHAB, BRCA1, CDKN1A and GADD45A) were analyzed by Pearson’s and Spearman’s correlation (P and Sp, respectively). Only the Survivin-RPRM gene pair showed a tendency toward mutual exclusion (shown in red), while other gene pairs showed a positive correlation (shown in green). (-) Gene pairs with a Pearson’s or Spearman’s coefficients $-0.3 \leq r$ or $\rho \leq 0.3$.

See Supplementary File 2

Supplementary Table 3: Cases included for linear regression analysis. Survivin and RPRM counts and p53 mutational status. Two-hundred and thirty-seven gastric cancer cases met the criteria: Available p53 status (from cBioPortal) and counts greater than 0 for both Survivin and RPRM. One hundred and twenty-one cases have wild-type p53, and one hundred and sixteen cases have mutated p53.

See Supplementary File 3

Supplementary Material: R code for manual retrieval from cBioPortal and cross-referenced to the RNAseq expression matrix using the unique TCGA identifiers (barcode) for each case.

See Supplementary File 4
